# Recent design strategies for boosting chemodynamic therapy of bacterial infections

**DOI:** 10.1002/EXP.20230087

**Published:** 2023-10-17

**Authors:** Junjie Zhang, Haiyang Guo, Ming Liu, Kaiyuan Tang, Shengke Li, Qiang Fang, Hengda Du, Xiaogang Zhou, Xin Lin, Yanjun Yang, Bin Huang, Dongliang Yang

**Affiliations:** ^1^ School of Fundamental Sciences Bengbu Medical College Bengbu China; ^2^ Macao Centre for Research and Development in Chinese Medicine Institute of Chinese Medical Sciences University of Macau Taipa Macau SAR China; ^3^ Anhui Key Laboratory of Infection and Immunity, School of Basic Medicine Bengbu Medical College Bengbu China; ^4^ School of Optometry and Ophthalmology and Eye Hospital, State Key Laboratory of Optometry Ophthalmology and Vision Science Wenzhou Medical University Wenzhou Zhejiang China; ^5^ School of Electrical and Computer Engineering, College of Engineering The University of Georgia Athens Georgia USA; ^6^ Academy of Integrative Medicine, Fujian Key Laboratory of Integrative Medicine on Geriatrics Fujian University of Traditional Chinese Medicine Fuzhou Fujian China; ^7^ Key Laboratory of Flexible Electronics (KLOFE) & Institute of Advanced Materials (IAM), School of Physical and Mathematical Sciences Nanjing Tech University (NanjingTech) Nanjing China

**Keywords:** antibacterial, chemodynamic therapy, combined therapy, nanomaterials

## Abstract

The emergence of drug‐resistant bacteria poses a significant threat to people's lives and health as bacterial infections continue to persist. Currently, antibiotic therapy remains the primary approach for tackling bacterial infections. However, the escalating rates of drug resistance coupled with the lag in the development of novel drugs have led to diminishing effectiveness of conventional treatments. Therefore, the development of nonantibiotic‐dependent therapeutic strategies has become imperative to impede the rise of bacterial resistance. The emergence of chemodynamic therapy (CDT) has opened up a new possibility due to the CDT can convert H_2_O_2_ into •OH via Fenton/Fenton‐like reaction for drug‐resistant bacterial treatment. However, the efficacy of CDT is limited by a variety of practical factors. To overcome this limitation, the sterilization efficiency of CDT can be enhanced by introducing the therapeutics with inherent antimicrobial capability. In addition, researchers have explored CDT‐based combined therapies to augment its antimicrobial effects and mitigate its potential toxic side effects toward normal tissues. This review examines the research progress of CDT in the antimicrobial field, explores various strategies to enhance CDT efficacy and presents the synergistic effects of CDT in combination with other modalities. And last, the current challenges faced by CDT and the future research directions are discussed.

## Graphical abstract

In this review, the recent progress of CDT in the antimicrobial therapy has been introduced. Then the strategies to boost the therapeutic effect of CDT and the CDT‐based combined therapeutic strategies are outlined. Finally, the challenges and opportunities of CDT are also discussed.

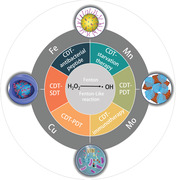

## 1 INTRODUCTION

Bacterial infection is a series of pathological processes caused by bacterial invasion, proliferation and interaction with the host.^[^ ^1^ ^]^ The growth and reproduction of bacteria in the host can induce many adverse effects toward the host, such as delayed wound healing,^[^ ^2^ ^]^ sepsis,^[^ ^3^ ^]^ and subcutaneous abscesses.^[^ ^4^ ^]^ In addition, bacterial infections can inhibit the production of red blood cells and affect the treatment of anemia.^[^ ^5^ ^]^ Some recent studies have also shown that severe bacterial infections can also affect the neurological development of newborns.^[^ ^6^ ^]^ The traditional treatment for bacterial infections is the use of antibiotics, represented by cephalosporins and penicillins.^[^ ^7^ ^]^ However, conventional antibiotic therapy will gradually screen out the bacteria with antibiotic resistance, eventually leading to an increased bacterial resistance and the emergence of multidrug resistant bacteria.^[^ ^8^ ^]^ The progressive growth of bacterial resistance has threatened human health and the economic development of society. For example, multidrug resistant bacteria have caused more than 700,000 deaths worldwide each year.^[^ ^9^ ^]^ Currently, antibacterial nanomaterials are gaining increasing attention due to their high targeting, high biocompatibility, and excellent antibacterial properties. For example, metal nanomaterials, carbon‐based nanomaterials, organic‐inorganic composite nanomaterials, etc., have been widely investigated.^[^ ^10^ ^]^ Nanomaterials execute their antibacterial activity mainly through the following pathways: (1) Some nanomaterials with a positive charge can interact with negatively charged bacterial membranes to precisely deliver antibacterial drugs to the infected site.^[^ ^11^ ^]^ (2) Some nanomaterials with Fenton‐like catalytic activities (i.e. chemodynamic agents) can decompose H_2_O_2_ in the biofilm microenvironment (BME) and generate highly oxidative reactive oxygen species (ROS) for sterilization purpose.^[^ ^12^ ^]^ (3) Nanomaterials can directly lyse biofilms, allowing the interaction between the bacteria within biofilms and antibacterial drugs.^[^ ^13^ ^]^ (4) The photosensitizers, sonosensitizers, and photothermal agents can be delivered to the infected site to trigger photodynamic therapy (PDT), sonodynamic therapy (SDT), photothermal therapy (PTT) and even combined treatment.^[^ ^14^ ^]^ Among various nanomaterial‐based antimicrobial therapies, chemodynamic therapy (CDT) is considered an effective antimicrobial strategy. CDT was used in the treatment of tumours at an early stage.^[^ ^15^ ^]^ Due to the fact that infection microenvironment shares many similarities with tumour microenvironment, such as overexpression of glutathione (GSH) and H_2_O_2_, and acidic pH, scientists have introduced it into bacterial infection treatment.^[^ ^16^ ^]^ And the similarity and difference between infection and tumour microenvironment can be seen in the previous reviews.^[^ ^17^ ^]^

CDT is a therapeutic method based on the Fenton/Fenton‐like reaction that decomposes H_2_O_2_ to produce hydroxyl radicals (•OH) and is widely used in the anticancer and antibacterial fields. The typical reaction equation of CDT is Fe^2+^ + H_2_O_2_ → Fe^3+^ + •OH + OH^−^.^[^ ^18^ ^]^ CDT mainly relies on highly toxic •OH to kill bacteria. First, •OH can effectively shear the extracellular DNA (eDNA) in the extracellular polymeric substances (EPS), thus disrupting the bacterial biofilm and exposing the bacterial cells buried in the biofilm. The highly toxic •OH then directly contacts the bacterial cell, destroying the lipids, proteins and other substances in the bacterial membrane, causing cell membrane rupture and leakage of bacterial cell contents, further resulting in bacterial death.^[^ ^19^ ^]^ CDT is a highly H_2_O_2_‐dependent treatment that can act specifically in the BME, where the H_2_O_2_ concentrations are relatively high. At the same time, the CDT‐based antibacterial strategy is not affected by bacterial resistance and may serve as an excellent nonantibiotic therapeutic method.^[^ ^20^ ^]^ However, the efficacy of CDT is also limited by a variety of practical factors. Specifically, (1) the conditions of the Fenton reaction are demanding. In general, the pH required for CDT is low, with an optimum pH of approximately 4. However, at the infection site, most infected tissues have a weakly acidic pH, and under these conditions, most Fenton reactions cannot undergo effectively.^[^ ^21^ ^]^ (2) Although the content of H_2_O_2_ at the infection site is relatively high compared to normal tissue, the concentration of H_2_O_2_ at the infection site still needs to be increased because low concentrations of H_2_O_2_ are detrimental to the generation of •OH, even with the presence of effective catalysts.^[^ ^22^ ^]^ (3) The GSH overexpression at the infection site consumes the •OH generated by the Fenton reaction, which will weaken the antibacterial effect of CDT.^[^ ^23^ ^]^ These aforementioned problems are major obstacles to the clinical application of CDT, so it becomes important to find a suitable solution. In addition to unlocking the limiting factors of CDT, the use of CDT in combination with other antimicrobial therapies is also a hot research topic. CDT is now widely used in combination with other therapeutic strategies. Examples include CDT‐PTT, CDT‐PDT, CDT‐SDT, CDT‐starvation therapy, CDT‐antibacterial peptides, and CDT‐immunotherapy. Abundant evidences confirm that combining CDT with other antimicrobial therapies can achieve better therapeutic results.^[^ ^24^ ^]^

**FIGURE 1:**
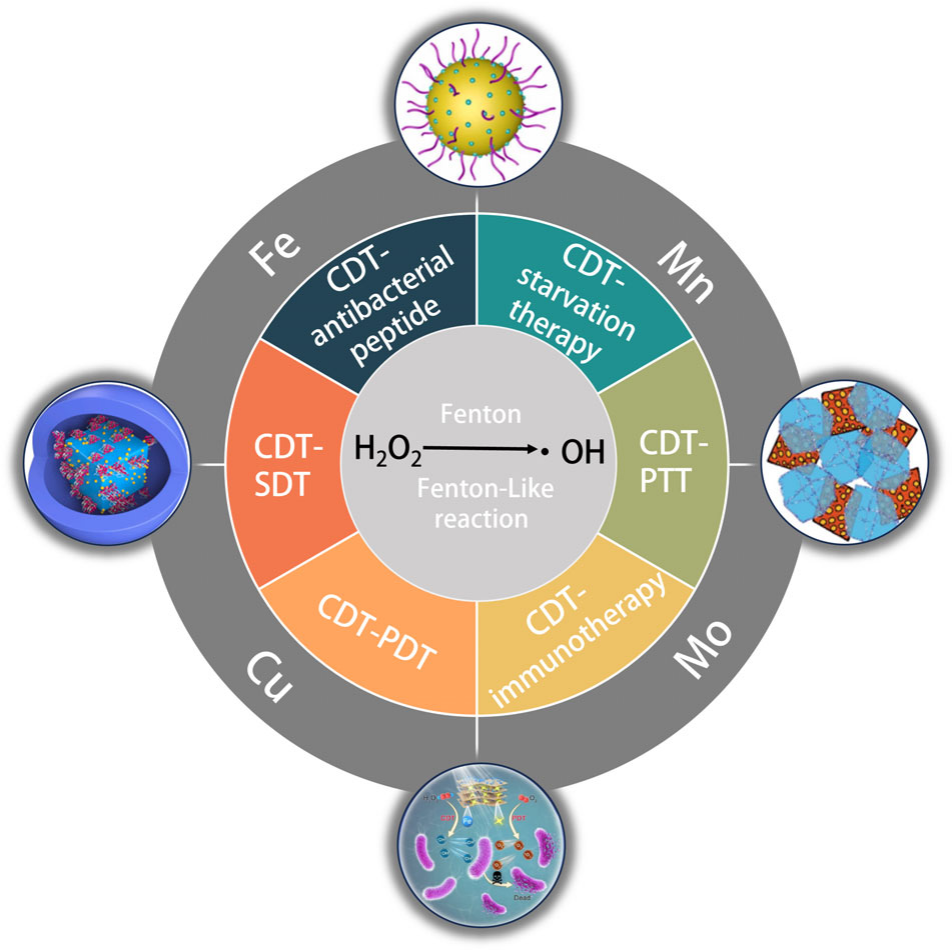
Schematic diagram of CDT‐based combination therapy.

Although CDT is an emerging treatment strategy, the development of CDT is progressing rapidly. Currently, CDT has become a popular direction in antimicrobial research.^[^ ^25^ ^]^ Previously, some reviews about CDT therapeutic platforms as well as combination therapies of CDT with other therapies have been summarized for cancer therapy.^[^ ^26^ ^]^ In this review, we will introduce the principles of CDT antimicrobial therapy and the potential strategies to improve the efficacy of CDT based on the infection microenvironment, including reducing the pH value of the infection site, inhibiting GSH overexpression, and increasing the H_2_O_2_ content at the site of infection. Then, the combination of CDT with other antibacterial therapies (e.g., PTT, PDT, SDT, starvation, antimicrobial peptides, immunization, and ferroptosis) is outlined to improve the therapeutic efficacy of CDT in the antimicrobial field (Figure 1). Finally, the bottleneck and opportunity of CDT are also discussed.

### 1.1 CDT therapy

**FIGURE 2:**
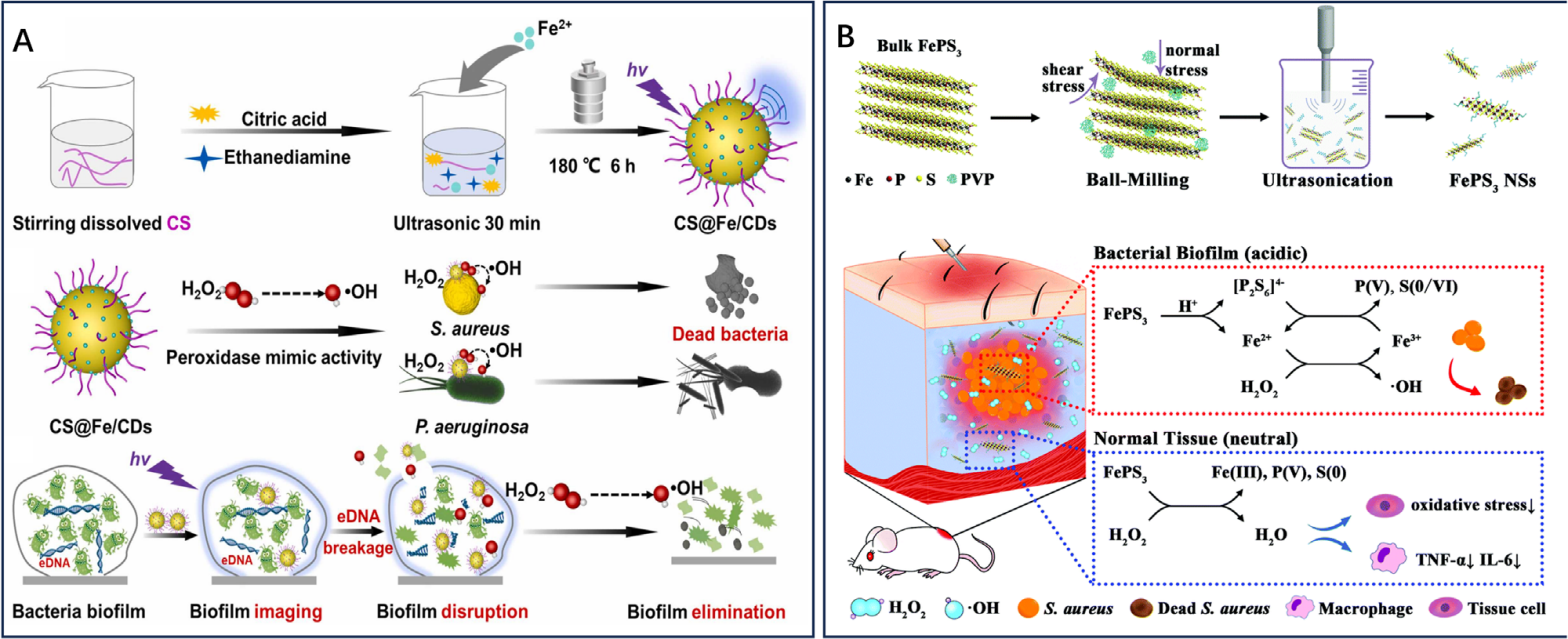
CDT‐mediated antibacterial therapy. A CS‐modified Fe‐doped carbon dots (CS@Fe/CDs) for disruption of bacterial biofilms. Reproduced with permission.^[^ ^19^ ^]^ Copyright 2022, Elsevier Ltd. B FePS_3_ nanoparticles for nonantibiotic antibacterial treatments. Reproduced with permission.^[^ ^32^ ^]^ Copyright 2021, The Royal Society of Chemistry.

Bacterial infections are usually treated with antibiotics, but in recent years, the misuse of antibiotics has led to an increase in antibiotic resistance. To combat drug‐resistant “superbugs”, the dose of antibiotics is increased, novel antibiotics are developed, and multimodal combination therapy is conceived.^[^ ^8^, ^27^ ^]^ However, the above methods do not solve the substantial problem of antibiotic resistance, and the search for a nonantibiotic treatment strategy can provide a new route to solve this problem.^[^ ^28^ ^]^ CDT is considered an effective antimicrobial treatment because of its high selectivity, low side effects, and nonantibiotic dependence.^[^ ^24d^ ^]^ CDT relies on Fenton/Fenton‐like reactions to produce highly toxic •OH by decomposition of H_2_O_2_, which can destroy bacterial components (e.g., nucleic acids and proteins), thereby killing pathogens and inhibiting bacterial growth and reproduction.^[^ ^29^ ^]^ However, the bacterial biofilm can isolate the bacteria from the external environment, forming a physical barrier that protects the bacteria from exogenous antibacterial substances such as antibiotics and antimicrobial peptides, and the bacterial biofilm can also induce the formation of an immunosuppressive chemical barrier.^[^ ^30^ ^]^ To overcome the biofilm barrier, Pan et al. used chitosan (CS), citric acid, ethylenediamine, and FeSO_4_‐7H_2_O as raw materials to prepare CS‐modified Fe‐doped carbon dots (CS@Fe/CDs) by a one‐pot hydrothermal method (Figure 2A).^[^ ^19^ ^]^ As a chemodynamic agent with peroxidase activity, CS@Fe/CDs could selectively destroy eDNA, further disrupting bacterial biofilms. First, the Fe doped in CS@CDs can trigger the Fenton‐like reaction and catalyse the decomposition of H_2_O_2_ to produce •OH to destroy eDNA in EPS, thus effectively cleaving the bacterial biofilm. On this basis, CS‐modified Fe/CDs with a positive charge can interact with negatively charged bacterial cell membrane through electrostatic interactions and exert their sterilizing effect by destroying membrane permeability and interfering the synthesis of DNA/RNA. CS@Fe/CDs have tremendous application prospects in the field of nonantibiotic sterilization due to their inexpensive raw material and strong sterilization effect.^[^ ^31^ ^]^ The •OH produced by CDT has antibiofilm activity, but at the same time, the highly toxic •OH also has a killing effect on normal tissues. Therefore, it is necessary to enhance the production performance of •OH and reduce its damage to normal tissues in clinical practice. Our group used iron powder, sulfur powder, and phosphorus powder to produce bulk FePS_3_ by a high‐temperature solid‐phase reaction, and then FePS_3_ nanoparticles (FePS_3_ NSs) were prepared by ball milling and liquid sonication (Figure 2B).^[^ ^32^ ^]^ Meanwhile, polyvinylpyrrolidone (PVP) polymer was used to improve the stability of FePS_3_ NSs. Bacteria in an oxygen‐deficient environment undergo anaerobic respiration and produce acidic metabolites, resulting in a low pH in the infected region. Thus, in the infected tissue, FePS_3_ NSs are decomposed to Fe^2+^ and [P_2_S_6_]^4−^.The resulting Fe^2+^ implements the Fenton reaction to form •OH while itself is oxidized to Fe^3+^. In the absence of exogenous reducing agents, the rate of Fe^3+^ reduction to Fe^2+^ is slower than the rate of Fe^2+^ oxidation, and this unbalanced Fe^2+^/Fe^3+^ cycle makes the supply of Fe^2+^ insufficient and slows the Fenton reaction rate. Interestingly, the [P_2_S_6_]^4−^ generated by the decomposition of FePS_3_ NSs under acidic conditions can be used as a reducing agent to promote the conversion of Fe^3+^ to Fe^2+^, which maintains a high Fenton reaction rate at the infected site and generates a large amount of •OH to achieve bactericidal purposes. In neutral normal tissues, FePS_3_ NSs exhibited antioxidant activity and could effectively scavenge H_2_O_2_ and •OH to minimize oxidative damage. In addition, H_2_O_2_ and •OH also act as secondary cellular messengers of inflammatory cytokines. The inflammatory reaction can be relieved by removing ROS. Therefore, using FePS_3_ NS‐based therapeutics, the harmful effects of •OH generated during CDT on normal tissues could be minimized, and the Fenton‐like catalytic activity reaction could be specifically activated in infected tissues, which possess potent application promise in anti‐infective therapy.

## 2 METAL IONS‐MEDIATED FENTON REACTION

In addition to the common iron and copper ions, other variable valence metal ions can be used for the CDT of bacterial infections.^[^ ^33^ ^]^ For example, the interconversion between Mn^2+^ and Mn^4+^ can catalyse the degradation of H_2_O_2_. Based on this result, Wang et al. prepared PLNPs@MSN@CA‐HA‐MnO_2_ (PMC‐HA‐MnO_2_) for the treatment of bacterial infections by modifying hyaluronic acid (HA) and MnO_2_ shells onto the surface of nanoparticles after mesoporous silica‐coated persistent luminescent nanoparticles (PLNPs@MSN) were loaded with cinnamic aldehyde (CA). Among this nanoplatform, CA is an alternative to antibiotics with a wide range of bactericidal capabilities. Mesoporous silica (MSN)‐coated PLNPs (PLNPs@MSN) have many pores, which can be used for drug delivery. The MnO_2_ shell could be degraded by the H_2_O_2_ that is located in the infected tissue, resulting in the production of Mn^2+^ for the Fenton catalytic reaction and the fluorescence recovery of PLNPs for the imaging of infected tissue. Using PMC‐HA‐MnO_2_ NPs, the authors could realize imaging‐guided on‐demand CDT/antibiotic therapy.^[^ ^34^ ^]^

Molybdenum is another common variable valence element. Based on its normality nature, Dong et al. developed an injectable hydrogel for silver/photothermal/chemodynamic antibacterial therapy. This hydrogel could be formed after urea was added to the mixture of tea polyphenols, gelatin, and Ag‐doped Mo_2_C‐derived polyoxometalate (AgPOM). After this hydrogel being applied to the infected wound, the hydrogel could prevent the wound from being damaged or infected again. At the same time, the photothermal capacity of AgPOM could be induced under acidic conditions for photothermal antibacterial therapy. The Mo^5+^ in the AgPOM could generate ROS for CDT. The antibacterial experiments indicated that this hydrogel with silver, CDT, and PTT therapeutic performances could kill the bacteria effectively, further promoting wound healing rapidly.^[^ ^35^ ^]^

In addition to the metal ions mentioned above, other emerging elements for CDT were summarized in the Jia's review.^[^ ^36^ ^]^

## 3 WAYS TO IMPROVE THE EFFICACY OF CDT

Due to the limitations of CDT, its antimicrobial activity is limited to some extent. Therefore, improving the therapeutic efficacy of CDT is a matter of great urgency. In recent years, researchers have explored several methods to enhance antimicrobial efficacy, such as (1) reducing the pH value of the infection site; (2) inhibiting the overexpression of GSH; and (3) increasing the H_2_O_2_ content at the infection site.

### 3.1 Reducing the pH value of the infection site

**FIGURE 3:**
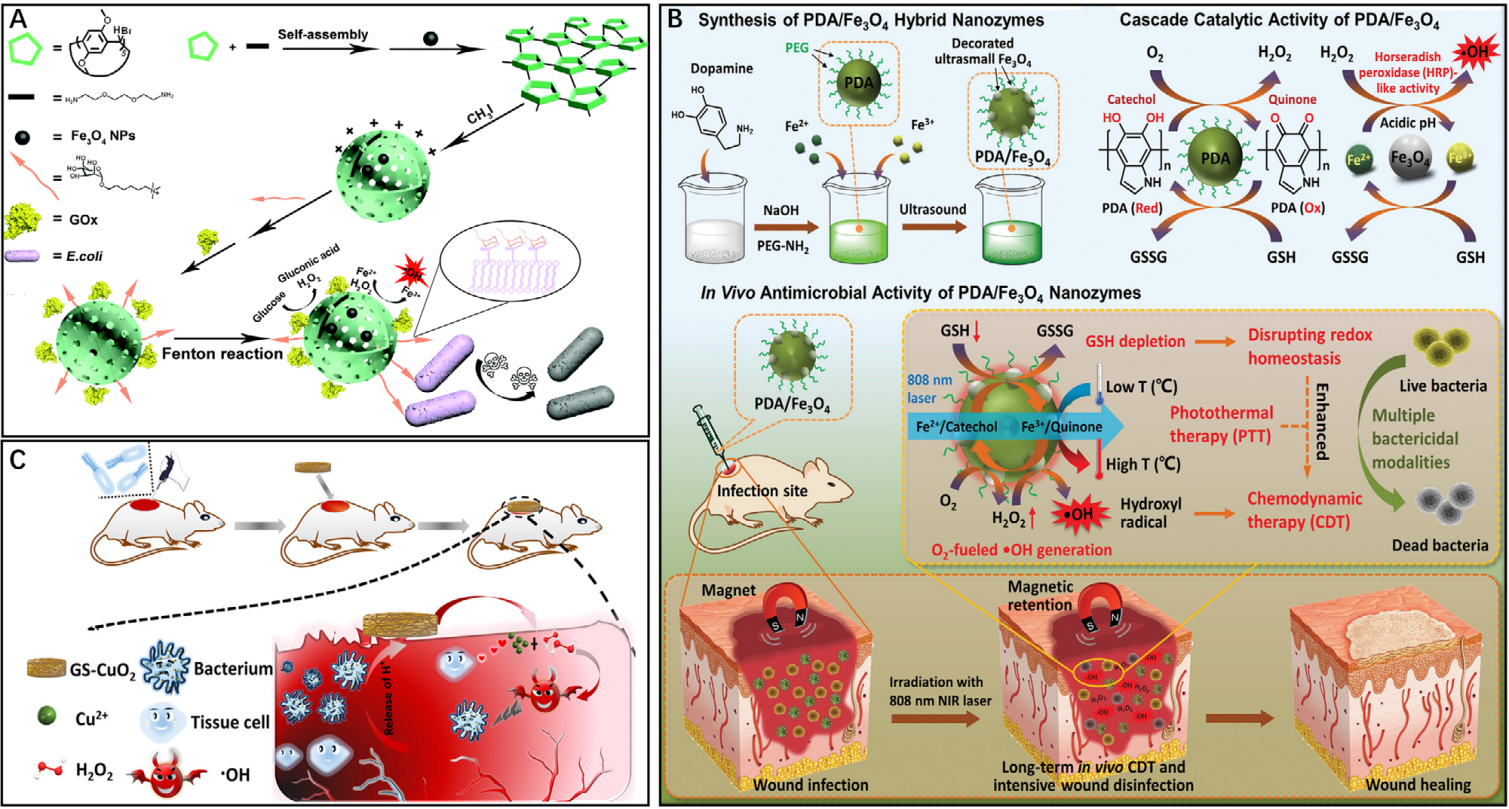
The strategies to boost the therapeutic effect of CDT. A Gox‐NCs/Fe_3_O_4_ for enhancing the antibacterial activity of CDT by acidized infected tissue and in situ self‐supplying H_2_O_2_. Reproduced with permission.^[^ ^39^ ^]^ Copyright 2022, The Royal Society of Chemistry. B PDA/Fe_3_O_4_ composite with photothermal‐enhanced cascade catalytic and GSH depletion performances for the long‐term removal of wound bacteria. Reproduced with permission.^[^ ^48^ ^]^ Copyright 2021, John Wiley & Sons, Inc. C GS‐CuO_2_ NPs for the treatment of wound infection. Reproduced with permission.^[^ ^43^ ^]^ Copyright 2022, ACS Publications.

The pH value in the infected environment is weakly acidic.^[^ ^37^ ^]^ The pH value is close to a neutral environment, which is detrimental to the occurrence of the Fenton reaction and reduces the antimicrobial efficiency of CDT.^[^ ^38^ ^]^ The antimicrobial efficiency of CDT can be effectively improved by reducing the pH value of the infected tissue. Li et al. constructed a cascade catalytic nanoplatform (Gox‐NCs/Fe_3_O_4_) by wrapping Fe_3_O_4_ nanoparticles with covalently assembled pillararene‐based polymer capsules (NCs) and then adsorbing glucose oxidase (Gox) onto its surface (Figure 3A).^[^ ^39^ ^]^ In the presence of glucose, Gox preloaded onto Gox‐NCs/Fe_3_O_4_ can effectively catalyse the production of H_2_O_2_ and glucuronic acid from glucose. Thus, as the concentration of Gox‐NCs and glucose increased, the pH value decreased. When the pH value drops below 4, the Fenton reaction will proceed efficiently, thus enhancing the therapeutic effect of CDT at the infected site. Similarly, to solve such problems, Dong et al. prepared Cu_2_O/Pt nanocubes and GOx‐doped alginate (ALG)‐hyaluronic acid (HA) hydrogels (Cu_2_O/Pt hydrogels). At the site of bacterial infection, GOx catalyses glucose to produce gluconic acid and H_2_O_2_, reducing the pH of the infected site while consuming glucose. After the pH decrease, it is conducive to the Fenton reaction, which in turn enhances CDT efficacy.^[^ ^40^ ^]^ However, reshaping the pH value at the disease site remains a challenge in clinic.

### 3.2 Inhibition of GSH overexpression

In infected tissue, the overexpression of GSH can clear •OH, which substantially reduces the antimicrobial efficacy of CDT.^[^ ^41^ ^]^ Therefore, it is urgent and necessary to reduce the GSH level at the bacterial infection site.^[^ ^42^ ^]^ Xiao et al. synthesized a novel chemodynamic agent (PDA/Fe_3_O_4_) by modifying polydopamine (PDA) with ultrasmall Fe_3_O_4_ (Figure 3B).^[^ ^43^ ^]^ This chemodynamic agent could consume the GSH overexpressed at the infection site to disrupt cellular redox homeostasis, while PDA/Fe_3_O_4_ with H_2_O_2_ self‐supplying capability could improve the production of •OH, which enhances the efficacy of CDT in wound disinfection. In this system, the PDA/Fe_3_O_4_ hybrid not only directly destroys bacteria but also enhances the horseradish peroxidase‐like activity. Similarly, Li et al. loaded Gox and a thermally unstable azo initiator, water‐soluble 2,2′‐azobis[2‐(2‐imidazolin‐2‐yl)propane] dihydrochloride (AIBI) onto flower‐like MnO_2_ to construct a dual radical nanogenerator (MnO_2_/Gox/AIBI) for reducing GSH overexpression at the infection site to enhance the efficacy of CDT.^[^ ^44^ ^]^ On the one hand, MnO_2_ can deplete overexpressed GSH at the infection site; on the other hand, MnO_2_/Gox/AIBI can use glucose to induce a cascade catalytic reaction to generate •OH for bacterial elimination because Gox can catalyse glucose to produce H_2_O_2_ and gluconic acid, which exhibits strong application prospects in diabetic wound infection. Another example was presented by Li et al. In their work, a multifunctional chemodynamic agent (MoS_2_/CuO_2_) was constructed by decorating ultrasmall CuO_2_ nanodots onto a hydrangea‐like MoS_2_ nanocarrier using a covalent Cu─S bond. This multifunctional agent (MoS_2_/CuO_2_) can regulate oxidative stress levels by using multivalent transition metal ions (Cu^2+^ and Mo^6+^) to deplete the overexpressed GSH at the injection site. In the acidic infected site, CuO_2_ on the surface of MoS_2_ decomposes and produces H_2_O_2_, and then H_2_O_2_ can be converted to •OH for bacterial inactivation due to the peroxidase‐mimicking activity of MoS_2_. By combining GSH depletion and H_2_O_2_ self‐supplying strategies, the therapeutic performance of CDT could be significantly enhanced.^[^ ^45^ ^]^

### 3.3 Increasing the H_2_O_2_ content at the infection site

Insufficient H_2_O_2_ at the site of bacterial infection makes the antimicrobial efficacy of CDT unsatisfactory, but excessive H_2_O_2_ can also cause irreversible damage to DNA, proteins, and other biomacromolecules in normal tissue.^[^ ^46^ ^]^ Therefore, it is necessary and urgent to develop a new catalytic agent to enhance the antimicrobial efficacy of CDT by modestly increasing the H_2_O_2_ content at the infection site. Wu et al. developed a CaO_2_@ZIF‐67‐PLL composite nanogenerator to improve the efficacy of CDT at bacterial infection sites.^[^ ^47^ ^]^ In an acidic environment, ZIF‐67 can rapidly decompose, and the Co^2+^ and CaO_2_ components are released. CaO_2_ reacts with water to produce H_2_O_2_ and O_2_. The production of O_2_ improves the hypoxic environment at the injection site. The produced H_2_O_2_ is catalysed by Co^2+^ to produce •OH, further enhancing the antibacterial ability at the infected site. In addition, CaO_2_@ZIF‐67‐PLL nanogenerators can inhibit the inflammatory response and stimulate angiogenesis, making them potential for the treatment of infected wounds. Although the H_2_O_2_ deficiency at the infected site has greatly improved, the targeted delivery of H_2_O_2_ to the infected tissue remains a major challenge. By using copper oxide (CuO_2_) as an H_2_O_2_ precursor, Cui et al. prepared a pH‐responsive CuO_2_‐loaded wound dressing using a gelatin sponge, copper hydroxide, and H_2_O_2_ as raw materials (Figure 3C).^[^ ^48^ ^]^ In infected tissue, this material can release Cu^2+^ and H_2_O_2_ and react to produce •OH to kill the pathogen. In addition, the release of collagen from the gelatin sponge can promote collagen deposition and stimulate angiogenesis. Thus, CuO_2_‐based gelatin sponges can generate •OH in situ for targeted elimination of bacteria and enhancement of wound healing.

## 4 CDT‐BASED COMBINED THERAPY

### 4.1 CDT‐PTT

Photothermal agents can absorb NIR laser light to convert light energy into heat energy.^[^ ^49^ ^]^ Previous studies confirm that high temperatures can promote Fenton‐like catalytic reactions. Thus, the combination of CDT and PTT effectively increases the efficiency of the Fenton reaction to produce more •OH, making it more effective in killing bacteria.^[^ ^50^ ^]^ In addition, compared to the NIR I laser, the NIR‐II laser has a deeper tissue penetration depth and a higher maximum permissible exposure (MPE).^[^ ^51^ ^]^ Based on these advantages, many therapeutic systems that possess CDT and PTT activities have been developed. For example, Lv et al. designed a polyoxometalate‐based heterojunction (GdP_5_W_30_@WS_2_) to treat infected wounds. Under 808 nm laser exposure, the peroxidase (POD)‐like activity of GdP_5_W_30_@WS_2_ is enhanced because the photoelectrons that were transferred from WS_2_ to GdP_5_W_30_@WS_2_ are beneficial for the conversion of W^6+^ to W^5+^. Thus, the overexpressed H_2_O_2_ in the infected tissue can be effectively converted into •OH for bacterial clearance by NIR‐triggered W^6+^/W^5+^ redox cycling. Moreover, the W^6+^ in GdP_5_W_30_@WS_2_ with GSH exhaustion ability can ensure ROS generation and further realize PTT and CDT combined therapy. Due to the high GSH content in the biofilm, GdP_5_W_30_@WS_2_ can be used as a biofilm microenvironment‐triggered therapeutic for the treatment of biofilm‐infected diseases.^[^ ^52^ ^]^Since the heat can increase the catalytic reaction rate, further enhancing the therapeutic efficacy of CDT. Many chemodynamic agents with photothermal performance have been developed.^[^ ^53^ ^]^ For example, Sun et al. developed an FeS@lauramidopropyl betaine LAB‐35@Ti_3_C_2_ chemodynamic agent for NIR‐II PTT‐enhanced CDT. The obtained FeS@LAB‐35@Ti_3_C_2_ with high photothermal conversion performance (*η* = 65.1%) can effectively convert the light energy of the 808 nm laser into heat energy. Then, the heat generated from FeS@LAB‐35@Ti_3_C_2_ can promote peroxidase‐like catalytic activity, further enhancing the generation of •OH for the removal of Gram‐negative and Gram‐positive pathogens from infected tissue.^[^ ^54^ ^]^ Similarly, Li et al. developed a meteor‐shaped gold‐manganese oxide nanoparticle (Au‐MnO_x_) for PTT/CDT synergistic antibacterial therapy. In addition, the interaction of pathogens with Au‐MnO_x_ can be enhanced due to its spike‐like morphology, further increasing the contact area between Au‐MnO_x_ and the bacteria. Upon application to infected tissue, with the addition of a 1064 nm laser and H_2_O_2_, Au‐MnOx exhibits a satisfactory bactericidal outcome.^[^ ^55^ ^]^ Based on the pH difference of infected tissue, some researchers have designed new acid‐responsive nanomaterials. Shi et al. then developed an acidity‐sensitive W/Mo‐based polymetallic oxalate (POM) for PTT‐enhanced CDT. In this system, Mo^5+^ in the POM catalyses the formation of •OH from H_2_O_2_ at the infection site through a Fenton‐like reaction. Meanwhile, Mo^5+^ will be oxidized into Mo^6+^, and the generated Mo^6+^ consumes the overexpressed GSH, thus reducing the GSH content at the injection site. After applying POM to the wound, the POM clusters aggregate to form large particles with strong NIR‐II absorbance in the acidic infected tissue. Under 1060 nm laser irradiation, the hyperthermia produced by POM can enhance the catalytic rate of the Fenton‐like reaction, further enabling efficient photothermal amplification of CDT.^[^ ^50a^ ^]^ These results indicated that the combination of CDT and PTT can significantly enhance the Fenton‐like catalytic rate and the production of •OH to boost the therapeutic effect of CDT.

### 4.2 CDT‐PDT

**FIGURE 4:**
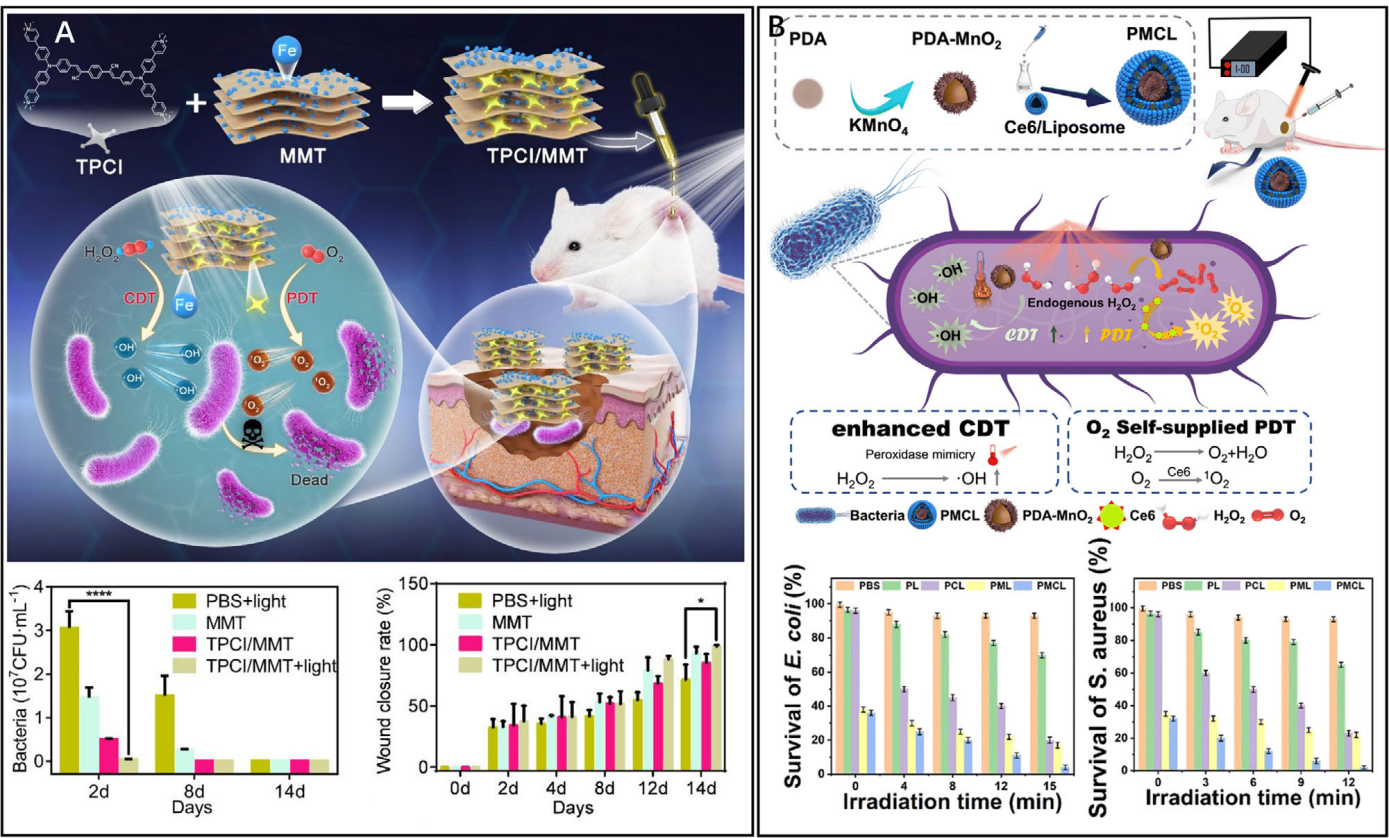
CDT‐PDT combined antibacterial therapy. A TPCI/MMT NPs for CDT‐PDT. Reproduced with permission.^[^ ^24b^ ^]^ Copyright 2022, ACS Publications. B PDA‐MnO_2_@Ce6/liposome for CDT‐PDT. Reproduced with permission.^[^ ^59^ ^]^ Copyright 2023, The Royal Society of Chemistry.

PDT is an antibiotic‐independent sterilization method in which a photosensitizer catalyses the generation of highly toxic ROS for the destruction of bacterial biofilms under light irradiation, thus causing the leakage of bacterial contents and bacterial death.^[^ ^56^ ^]^ However, there are some factors (e.g., insufficient oxygen content at the infection site and limited penetration depth of light) that limit the application of PDT.^[^ ^57^ ^]^ By combining CDT and PDT for sterilization, the limitations of PDT can be overcome while improving the sterilization effectiveness of CDT, which promotes the use of CDT in the antimicrobial field.^[^ ^58^ ^]^ And some representative combined examples are listed in Table 1. For example, Zhang et al. have developed an innovative theranostic platform (TPCI/MMT NPs) for efficient bacterial eradication and accelerating wound healing. TPCI/MMT NPs combine PDT and CDT and utilize fluorescence‐dependent visualization for infection detection. TPCI/MMT NPs were prepared by stirring an aqueous solution of polycationic polystyrene (TPCI) and iron‐containing montmorillonite (MMT) at room temperature. Through electrostatic and intermolecular interactions, TPCI was successfully loaded into the lamellar structure of MMT, resulting in the formation of TPCI/MMT. Then iron ions were subsequently loaded onto the surface, creating a combined CDT and PDT therapeutic platform for therapeutic diagnosis (Figure 4A).^[^ ^24b^ ^]^ Under white light irradiation, TPCI, acting as a photosensitizer, generates highly toxic singlet oxygen (^1^O_2_) in the infected microenvironment (IME) to disrupt the bacterial biofilm, cause bacterial content leakage and induce bacterial death. However, due to the limited penetration depth of exogenous light, photosensitizers in deep tissues cannot be activated to produce ^1^O_2_. In such cases, CDT serves as an alternative therapeutic method. At the infection site, the negatively charged bacterial biofilm absorbs the loaded Fe^3+^ ions from the surface of MMT, further promoting Fe^3+^ release. The Fe^3+^ ions within the biofilm catalyse the decomposition of endogenous H_2_O_2_ in the IME to generate •OH for the treatment of deep tissue bacterial infections. It is worth noting that the aggregation‐induced emission properties of TPCI enable the visual detection of TPCI/MMT in patients. In general, MMT can pose risks in clinical settings, as it tends to adhere to red blood cells and cause intravascular haemolysis. However, the insertion of TPCI into the interlayer of MMT alters the surface charge and aggregation pattern of MMT, reducing its adhesion to red blood cells and improving hemocompatibility. Similarly, TPCI may have side effects, such as photodamage to normal tissue cells, which limits its application. By inserting TPCI into the interlayer of MMT, the interaction between TPCI and normal cells can be minimized, reducing its side effects on normal tissue cells. This complementary effect enhances the biosafety of TPCI/MMT. Considering that the occurrence of PDT and CDT heavily relies on the concentrations of H_2_O_2_ and O_2_, maintaining optimal levels of both is crucial to maximize the therapeutic efficiency of PDT and CDT. However, in the IME, the contents of H_2_O_2_ and O_2_ are insufficient. Therefore, increasing the O_2_ and H_2_O_2_ concentrations at the infection site is vital to improve the therapeutic effect of CDT/PDT combined antimicrobial therapy. In this regard, a novel strategy was proposed by Ma et al. In this work, zeolite imidazole framework‐67 (ZIF‐67) was synthesized using methylimidazole and Co^2+^ and loaded with CaO_2_ and graphene quantum dots (GQDs) as a photosensitizer for bacterial biofilm clearance and activation of bacterial oxidative stress. Since the catabolism of ZIF‐67 is pH dependent, with a high rate of catabolism under acidic conditions and slow or almost no catabolism under neutral and basic conditions, this special property confers the ability of target drug delivery in acidic IMEs, where ZIF‐67 disintegrates itself to form Co^2+^ and release CaO_2_ and GQD. CaO_2_ can achieve endogenous delivery of H_2_O_2_ and O_2_ at the infection site by reacting with water to generate H_2_O_2_ and O_2_. Then H_2_O_2_ is further catalysed by Co^2+^ to form •OH to activate CDT. Similarly, the sufficient O_2_ supply at the infection site enhanced GQD‐mediated PDT by relieving the hypoxic environment, further achieving CDT and PDT dual enhancement. Therefore, CaO_2_/GQDs@ZIF‐67 with its high biosafety, targeting, and strong biofilm lysis abilities, can be used as a novel PDT/CDT combined antimicrobial agent.^[^ ^24a^ ^]^ It is worth noting that bacterial biofilms lysis caused by combined PDT/CDT may induce acute infection at the infection site and trigger a severe inflammatory response due to the release of a large number of free bacteria. Therefore, when using highly toxic •OH and ^1^O_2_ to remove recalcitrant biofilms, more attention should be given to the cleanup of biofilm fragments.^[^ ^24a^ ^]^ Shan's team designed a dual‐mode ROS generator based on a photosensitive chemodynamic agent (PDA‐MnO_2_@Ce6/liposome (PMCL)) by loading the PDA surface with rough MnO_2_ and finally wrapping it with the photosensitizer Ce6 and liposomes (Figure 4B).^[^ ^59^ ^]^ Among them, MnO_2_ can be used as a peroxidase to catalyse the decomposition of H_2_O_2_ to produce O_2_ to enhance the ^1^O_2_ production efficiency of PDT. Additionally, MnO_2_ can act as a peroxidase‐like enzyme that catalyses the generation of •OH from endogenous H_2_O_2_, thus triggering CDT. PDA as a photothermal agent is used to absorb exogenous NIR light, which can efficiently convert light energy into heat energy. Ce6 is used as a photosensitizer for PDT to catalyse the conversion of O_2_ to singlet oxygen, ^1^O_2_. Finally, the liposome was wrapped in the outermost layer and adhered to the bacteria by fusing with the bacterial membrane. This enables the targeted transport of PMCL, which to a certain extent avoids normal tissue damage due to MnO_2_ only works in infected areas and improves the biosafety and stability of PMCL. Notably, experimental studies showed that the biofilm clearance rate of *E. coli* treated with PMCL reached 95% under laser irradiation (671 nm, 0.48 W cm^−2^), indicating that PMCL had excellent biofilm eradication ability, which may be related to •OH‐induced M1‐type macrophage production and thus the removal of biofilm fragments. The combined CDT/PDT can overcome the inability of PDT to remove deep tissue bacteria. Meanwhile, with the introduction of a photosensitizer, the oxygen generated by H_2_O_2_ decomposition can not only be converted into ^1^O_2_ for bacterial biofilm clearance but also promote wound healing. Compared with CDT treatment alone, the combination of CDT and PDT has high antimicrobial efficiency and efficient use of endogenous H_2_O_2_, which is more conducive to the treatment of biofilm infection.

**TABLE 1:** Photosensitizers used for CDT/PDT combination therapy.

Nanoparticles	Photosensitizer	Performances	Ref.
TPCI/MMT NPs	TPCI	Inducing ^1^O_2_ generation under white light irradiation	[60]
CaO_2_/GQDs@ZIF‐67	Graphene quantum dots (GQDs)	Converting H_2_O_2_ and ^3^O_2_ into hydroxyl radicals and ^1^O_2_ under light irradiation	[24a]
PDA‐MnO_2_@Ce6/liposome (PMCL)	Ce6	Self‐supplying oxygen to enhance ^1^O_2_ generation	[59]
PLNP‐PS	Cyanine probe (PS)	Activable fluorescence imaging photodynamic therapy	[61]
ZIF‐ICG@ZIF‐GOX@MPN	Indocyanine green (ICG)	Inducing ^1^O_2_ generation under near‐infrared light irradiation	[21]
FeP@PL:ZnPc (COOH)_8_	ZnPc(COOH)_8_	GSH depletion, inducing ROS generation	[62]

### 4.3 CDT‐SDT

SDT is a cutting‐edge treatment that utilizes sonosensitizers to eliminate bacteria through the generation of a substantial quantity of ROS under low‐intensity ultrasound (US).^[^ ^63^ ^]^ The application of US induces a fascinating sonoluminescence effect, effectively activating the sonosensitizers and resulting in the formation of electron‐hole pairs. Following the transition from an excited state to a ground state, the sonosensitizers release energy that is harnessed to facilitate the production of ROS by interaction with O_2_ and H_2_O. Moreover, the collapse of the cavitation bubbles at the infection site generates a localized increase in temperatures, leading to the production of highly potent •OH, which serves as an effective agent in eradicating bacterial biofilms. Combining SDT with CDT exhibits remarkable synergistic effect by enhancing the production of ROS. In addition, SDT demonstrates exceptional tissue penetration, favourable biocompatibility, and specific targeting properties, which further contribute to its effectiveness.^[^ ^64^ ^]^ Therefore, the integration of SDT with CDT holds great promise for achieving enhanced therapeutic outcomes.^[^ ^65^ ^]^ Xin et al. developed a multifunctional nanotherapeutic platform for combating periodontitis through sonodynamic/chemodynamic combination therapy.^[^ ^24c^ ^]^ On this platform, dendritic mesoporous silica particles (DLMSNs) were utilized as a substrate for the growth of TiO_2_. Subsequently, Ag (DT‐Ag) was deposited onto the TiO_2_‐coated DLMSNs, and finally, quaternary ammonium chitosan was modified on the surface (DT‐Ag‐CS^+^). TiO_2_ serves as a stable inorganic sonosensitizer, generating electron (e^−^) and hole (h^+^) pairs when exposed to US. The released energy during the transition of TiO_2_ to the ground state catalyses the production of ROS from O_2_ and H_2_O, activating SDT. However, the ROS yield is limited when TiO_2_ is used alone against bacteria. To overcome this limitation, Ag NPs were modified onto the surface of TiO_2_ to trap excited electrons and prevent electron compounding, thereby promoting ROS production. Additionally, the introduction of Ag enhances the absorption value of nanomaterials, leading to improved SDT efficiency compared to TiO_2_ alone for antimicrobial treatment. Moreover, Ag^+^ not only disrupts the bacterial cell membrane, causing leakage of cytoplasmic contents and ultimately leading to bacterial death, but can also induce the Fenton reaction by interacting with endogenous H_2_O_2_, activating CDT. The positively charged quaternary ammonium group‐modified chitosan (CS) loaded on the nanotherapeutic platform interacts with the negatively charged bacterial biofilm, resulting in the cleavage of the biofilm and bacterial death. Similar to photosensitizers, sonosensitizers can exclusively induce the acoustic luminescence effect and activate the SDT when exposed to ultrasound. The delivery of sonosensitizers into the skin poses a significant challenge to the efficacy of SDT. By combining physical penetration methods, SDT becomes feasible to achieve dermal tissue sterilization. In recent studies, the integration of CDT with SDT has been investigated. For example, Song et al. designed a SDT/CDT combined therapeutic platform by incorporating Fe^3+^ ions into polyethyleneimine‐modified bismuth oxide (BiOBr) nanoplates.^[^ ^66^ ^]^ This sophisticated nanomaterial effectively facilitates the separation of electrons and holes on the surface of BiOBr under sound stimulation, thereby promoting efficient electron transfer and leading to the generation of a significant quantity of ROS. Concurrently, the presence of Fe^3+^ ions triggers a Fenton‐like reaction, resulting in the production of highly reactive •OH radicals. The combined generation of ROS and •OH radicals demonstrates a potent antibacterial effect. In addition, the incorporation of Fe^3+^ ions into this nanomaterial enables its use as a contrast agent for magnetic resonance imaging (MRI), enabling precise localization and accurate diagnosis of bacterial infection sites. These findings underscore the significant synergistic effect achieved by integrating CDT with SDT, which facilitates efficient ROS production through SDT while augmenting the performance of CDT, ultimately enhancing the effectiveness of bactericidal treatments.

### 4.4 CDT‐starvation therapy

**FIGURE 5:**
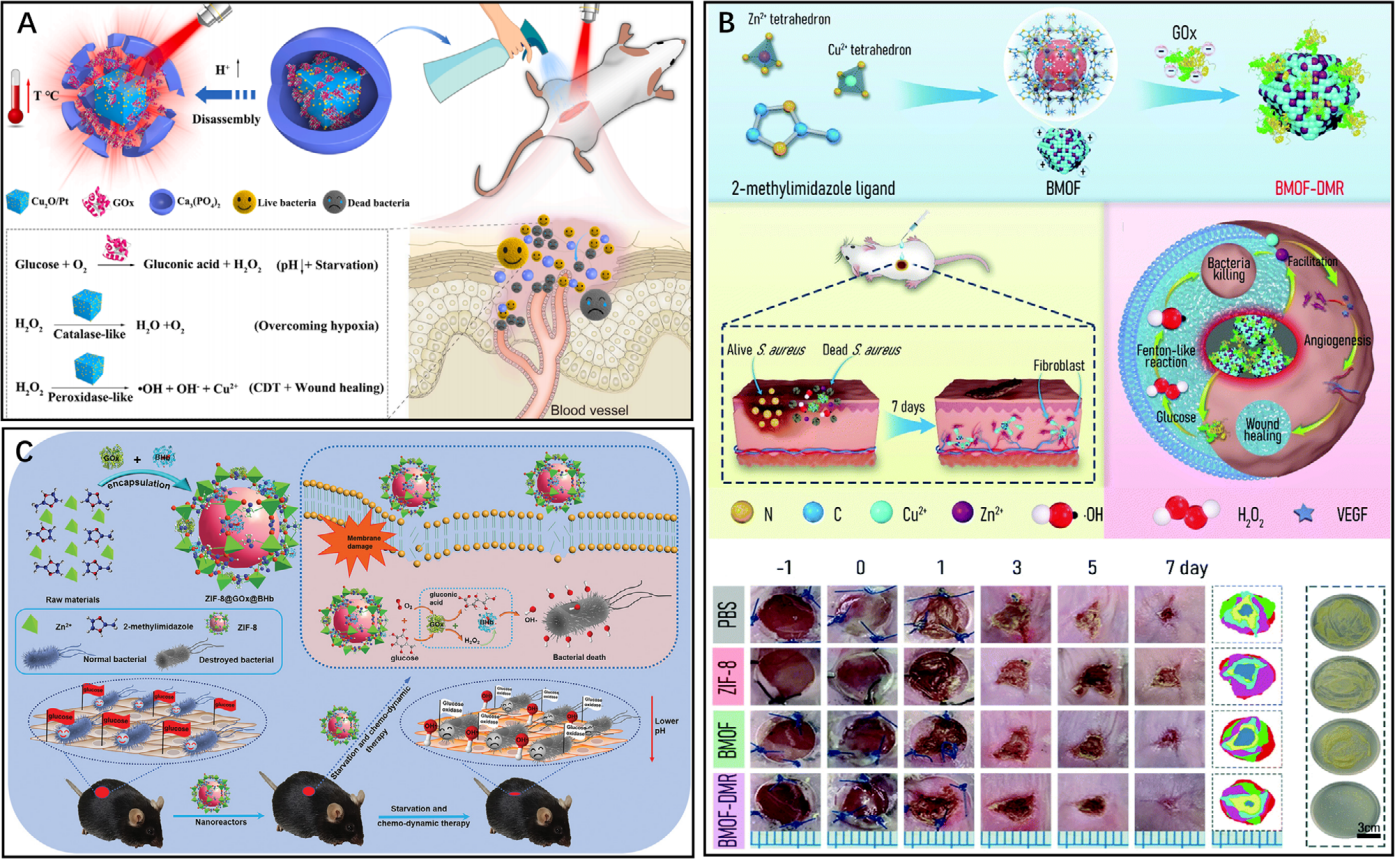
CDT‐starvation combined antibacterial therapy. A GOx‐CaPCuPt for CDT‐starvation therapy. Reproduced with permission.^[^ ^67^ ^]^ Copyright 2022, Elsevier Ltd. B BMOF‐DMR for CDT‐starvation therapy. Reproduced with permission.^[^ ^69^ ^]^ Copyright 2021, The Royal Society of Chemistry. C ZIF‐8@GOx@BHb combined with CDT and starvation therapy for lowering pH at the injection site. Reproduced with permission.^[^ ^70^ ^]^ Copyright 2021, John Wiley & Sons, Inc.

At the site of bacterial infection, glucose serves as a constant source of nutrients, supporting bacterial proliferation and growth. Starvation therapy can effectively diminish glucose levels at the infection site ^[^ ^67^ ^]^. However, the antimicrobial efficacy of starvation therapy alone is limited. To enhance its effectiveness, researchers have explored the combination of CDT with starvation therapy, aiming to achieve a more robust antimicrobial outcome. Wang et al. developed a novel core‐shell CaP‐Gox‐Cu2O/Pt (GOx‐CaPCuPt) nanoreactor for enhanced antibacterial therapy (Figure 5A).^[^ ^67^ ^]^ The synthetic process involved mineralizing Gox with Cu_2_O/Pt and subsequently encapsulating the product with calcium phosphate (CaP). This innovative nanoreactor utilizes Gox to effectively reduce glucose levels at the bacterial infection site, leading to significant inhibition of bacterial growth and proliferation. Furthermore, as glucose is consumed, H_2_O_2_ is generated, which is then converted to •OH and O_2_ by the catalysis of Cu_2_O/Pt. The generated O_2_ helps alleviate the hypoxic infection environment, while H_2_O_2_ is utilized in the Fenton reaction of CDT. Moreover, the production of gluconic acid leads to the acidification of the infected tissue, triggering the release of copper ions and consequently promoting angiogenesis. By combining starvation therapy with CDT, the GOx‐CaPCuPt nanoreactor demonstrates enhanced antibacterial efficacy and promotes wound healing. To maximize the antimicrobial potency of CDT and starvation therapy, Deng et al. devised a photoactivated cascade bioheterogeneity junctions (C‐bio‐HJs) to achieve rapid eradication of bacteria at the infection site.^[^ ^68^ ^]^ The bio‐HJs were loaded with Gox, enabling glucose oxidation for starvation therapy. Simultaneously, the H_2_O_2_ generated during glucose oxidation was catalysed by Mo^4+^ to produce •OH for CDT. By combining the cascade of bioheterogeneity junctions with CDT and starvation therapy, a synergistic antimicrobial effect was achieved.

In another study, Peng et al. developed a bimetallic metal‐organic framework domino microreactor (BMOF‐DMR) through electrostatic self‐assembly (Figure 5B).^[^ ^69^ ^]^ The BMOF‐DMR consists of a Zn/Cu‐rich BMOF and Gox. Gox consumes glucose at the infection site for starvation therapy. The produced H_2_O_2_ during glucose consumption is catalysed by Cu^2+^ to form •OH for CDT. A domino response is formed between these two processes. This nanomaterial not only depletes the source of bacterial nutrients but also stimulates the Fenton reaction and reduces the overexpressed GSH at the infection site, demonstrating efficient antibacterial and promoting wound healing capabilities in conjunction with CDT and starvation therapy. Furthermore, this nanomaterial exhibits excellent cytocompatibility, making it a promising approach for antimicrobial applications. Similarly, to combat multidrug‐resistant bacteria and accelerate diabetic wound healing, Li et al. developed an efficient cascade catalytic antibacterial system by immobilizing Gox and peroxidase‐like bovine hemoglobin (BHb) onto a metal‐organic nanostructured nanoreactor (Figure 5C).^[^ ^70^ ^]^ The metal‐organic frameworks (MOFs) possess a flexible void structure and a large surface area, providing an ideal protective environment for the loaded proteins against the influence of external factor . The incorporation of Gox and BHb into the MOFs led to the formation of ZIF‐8@GOx@BHb, which exhibited enhanced peroxide and Gox activity due to the spatial confinement. In this catalytic antimicrobial system, glucose consumption results in the production of gluconic acid and H_2_O_2_. With the presence of BHb, the generated H_2_O_2_ was efficiently converted into •OH for effective bacterial killing. Additionally, the production of gluconic acid resulted in an acidic pH in the affected area, thereby facilitating Fenton reactions. Moreover, ZIF‐8@Gox@BHb could be excreted from the body in fecal form. Therefore, this cascade catalytic nanoreactor system shows remarkable efficacy in bacterial eradication and holds great promise for clinical applications. In summary, the integration of CDT with starvation therapy not only effectively depletes the bacterial nutrient source but also generates H_2_O_2_, which is subsequently catalysed into •OH, thereby promoting CDT and leading to a pronounced synergistic effect.

### 4.5 CDT‐antibacterial peptide

**FIGURE 6:**
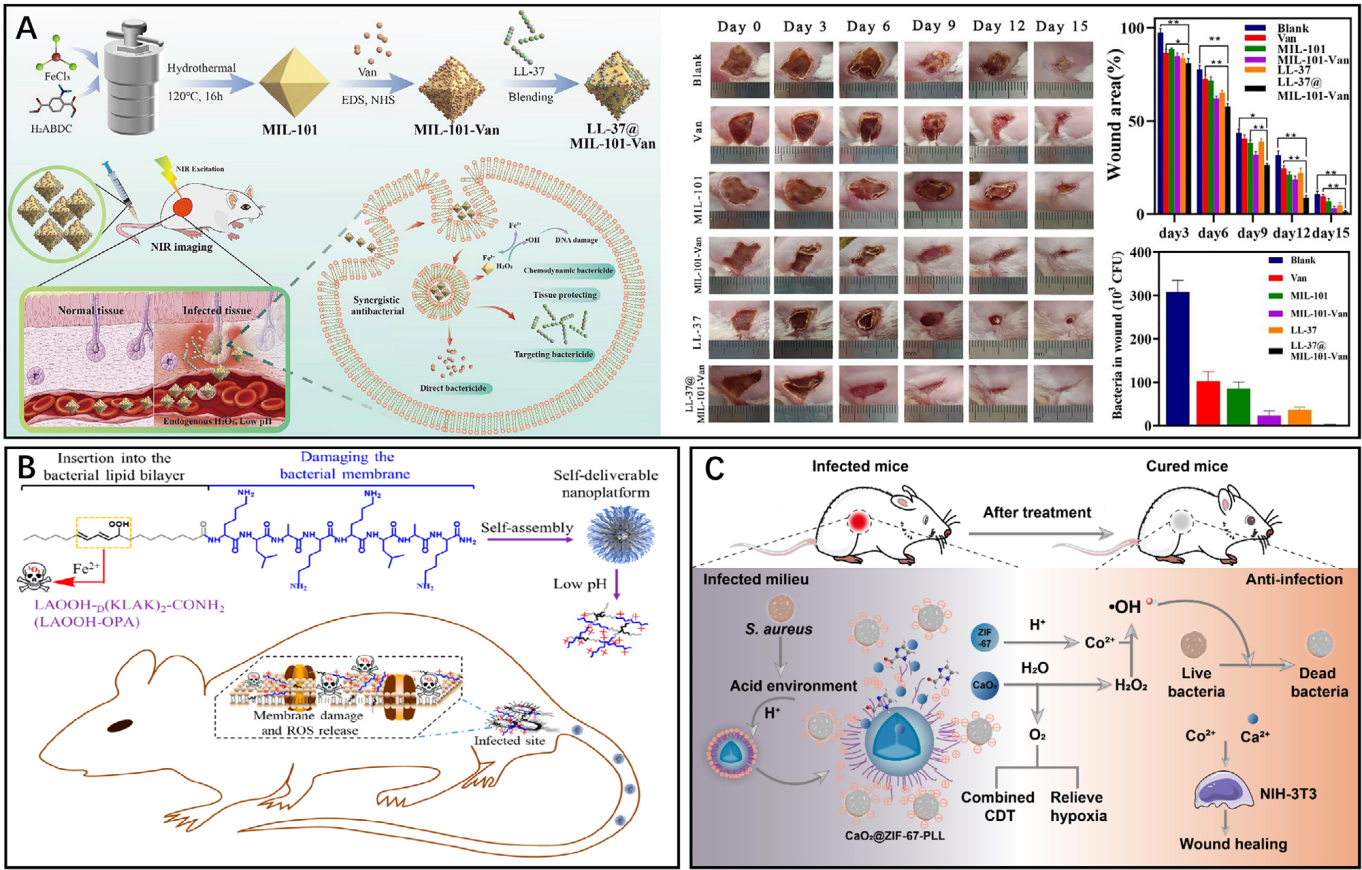
CDT‐antibacterial peptide combined antibacterial therapy: A LL‐37@MIL‐101‐Van for CDT‐AMPs. Reproduced with permission.^[^ ^73^ ^]^ Copyright 2022, Elsevier Ltd. B LAOOH‐OPA carrying D(KLAK)2 in combination with LAHP for CDT‐ AMPs. Reproduced with permission.^[^ ^74^ ^]^ Copyright 2021, ACS Publications. C CaO_2_@ZIF‐67‐PLL combined with CDT and AMPs. Reproduced with permission.^[^ ^47^ ^]^ Copyright 2023, Elsevier Ltd.

Antimicrobial peptides (AMPs) are potent agents with broad‐spectrum activity against various microorganisms, offering a promising approach for the treatment of bacterial infections while minimizing the development of bacterial resistance.^[^ ^27^, ^71^ ^]^ However, the efficacy of AMPs is hindered by several factors, such as pH sensitivity, high production cost, susceptibility to hydrolytic enzymes, among others.^[^ ^72^ ^]^ Consequently, there is a growing need to explore effective solutions to overcome these challenges and enhance the therapeutic potential of AMPs. Lai et al. developed a novel nanosystem, LL‐37@MIL‐101‐Van, to achieve synergistic treatment of drug‐resistant bacteria (Figure 6A).^[^ ^73^ ^]^ This nanosystem was synthesized by covalently linking vancomycin to MIL‐101 and subsequently modifying the surface with the targeted antimicrobial peptide LL‐37 which labelled with a near infrared fluorescent dye. Vancomycin can inhibit bacterial growth through interference with cell wall synthesis and altering cell wall permeability. Furthermore, LL‐37@MIL‐101‐Van exhibits excellent Fenton activity in the acidic microenvironment of infected sites, facilitating the decomposition of H_2_O_2_ and generation of •OH to effectively eliminate bacteria. Additionally, LL‐37 was labelled with a near‐infrared fluorescent dye to enable in vivo fluorescent imaging, providing a method to assess the therapeutic effects of LL‐37@MIL‐101‐Van. Moreover, AMPs possess a positive charge that allows them to interact with negatively charged bacterial membranes through electrostatic attraction. This interaction not only enhances the targeting of AMPs but also disrupts bacterial biofilms, leading to more effective sterilization. Based on this, Huang et al. developed a novel antimicrobial combination strategy by combining an amphiphilic oligopeptide (LAOOH‐OPA) carrying the therapeutic unit D(KLAK)2 with hydrophobic linoleic acid hydroperoxide (LAHP) (Figure 6B).^[^ ^74^ ^]^ The positively charged D(KLAK)2 with an α‐helical conformation enables rapid binding to microbial cells through electrostatic interactions and resulting in the inactivation of the bacterial cell membrane. In addition, upon activation of Fe^2+^ in the material, LAHP generates ^1^O_2_, leading to lipid bilayer leakage and bacterial inhibition. Meanwhile, the activation of Fe^2+^ also triggers the Fenton‐like reaction in CDT, leading to the production of abundant •OH, which ultimately kills bacteria. The combination of these two mechanisms not only generates •OH but also enhances the antimicrobial activity of the antimicrobial peptide, resulting in a stronger antimicrobial effect. Importantly, the pathway of lipid peroxidation utilizes linoleic acid to produce LAHP, serving as a source of ROS. The biological activity assessments confirm LAOOH‐OPA holds potent bactericidal effects and minimal toxic side effects. Using similar strategy, Wu and colleagues developed a novel nanocomposite, CaO_2_@ZIF‐67‐PLL, by encapsulating CaO_2_ within a cobalt metal‐organic framework (ZIF‐67) and simultaneously modifying its surface with hyperbranched poly‐L‐lysine (PLL) (Figure 6C).^[^ ^47^ ^]^ Previous research has demonstrated that PLL exhibits remarkable antibacterial properties through electrostatic interactions. Moreover, PLL with favorable cytocompatibility can penetrate the bacteria's cell membrane. Under normal physiological conditions, the material remains stable, preventing CaO_2_ from being hydrolysed prematurely. However, when exposed to a slightly acidic environment, this nanocomposite undergoes decomposition, releasing Co^+^ from the cobalt framework and revealing the CaO_2_ component. The reaction between CaO_2_ and H_2_O in turn generates H_2_O_2_ and O_2_. The released O_2_ alleviates the hypoxic microenvironment at the infection site, while the H_2_O_2_ triggers the Fenton reaction, resulting in the production of abundant •OH to effectively kill bacteria. Furthermore, PLL itself exhibits robust antimicrobial properties, further synergizing with CDT. Importantly, CaO_2_@ZIF‐67‐PLL demonstrates excellent biocompatibility, making it suitable for biomedical applications. The combination of CDT and AMPs represents a promising approach that can enhance the antimicrobial activity of AMPs, which has wider clinical application prospects. By incorporating AMPs into the CDT regimen, the antimicrobial efficacy can be further enhanced, offering a broader range of applications in clinical practice.

### 4.6 CDT‐immunotherapy

**FIGURE 7:**
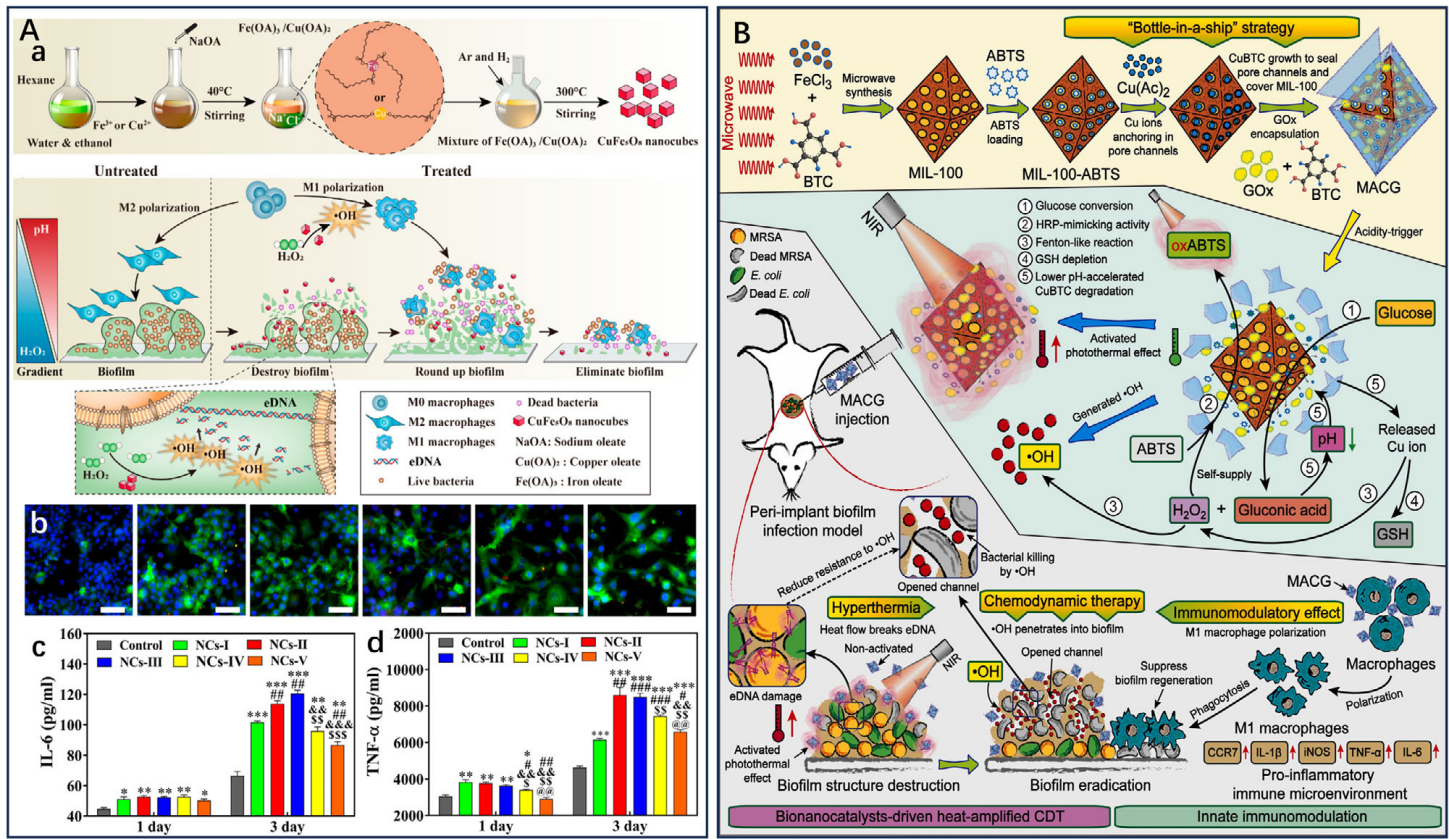
CDT‐immunotherapy combined antibacterial therapy. A CuFe_5_O_8_ NCs induce M1‐type macrophage differentiation for biofilm fragmentation and free bacterial clearance. The synthesis of CuFe_5_O_8_ NCs and its antibiofilm process a. CuFe_5_O_8_ NCs induce the polarization of M1‐type macrophages b and the secretion of pro‐inflammatory cytokines c,d. Reproduced with permission.^[^ ^30^ ^]^ Copyright 2020, ACS Publications. B Nanobionics catalyst MACG with bilayer structure for the reconstruction of pro‐inflammatory immune microenvironment. Reproduced with permission.^[^ ^77^ ^]^ Copyright 2023, Elsevier Ltd.

The BME plays a crucial role in promoting the differentiation of macrophages into the M2 subtype, which exhibits immunosuppressive effects and hinders the body's immune clearance of biofilms.^[^ ^75^ ^]^ This immunosuppression, coupled with the failure to promptly remove biofilms by •OH, can lead to severe acute infections at the infection site.^[^ ^76^ ^]^ Therefore, it is imperative to restore the normal immune response at the infection site to enhance the antibacterial efficiency of CDT. To address this challenge, chemodynamic agents with versatile functions have been developed. These chemodynamic agents not only degrade biofilms by generating toxic •OH but also reverse the immunosuppressive BME by activating immune cells, enabling direct bacterial killing. Consequently, the sterilizing effect of CDT is enhanced, and the occurrence of infections is prevented.^[^ ^29^, ^77^ ^]^ Based on this, Guo et al. developed a highly efficient catalyst, CuFe_5_O_8_ nanocubes (CuFe_5_O_8_ NCs), by pyrolyzing iron oleate [Fe(OA)_3_] and copper oleate [Cu(OA)_2_] precursors (Figure 7Aa).^[^ ^30^ ^]^ CuFe_5_O_8_ NCs demonstrate the ability to decompose H_2_O_2_ within bacterial biofilms, leading to the generation of abundant •OH, which facilitates the cleavage of extracellular DNA (eDNA) within the biofilms. In biofilm‐related infections, the failure to promptly remove biofilm fragments can result in the formation of an immunosuppressive microenvironment, leading to a severe acute infection in surrounding tissues. To overcome this challenge, CuFe_5_O_8_ NCs were employed to convert the low levels of H_2_O_2_ at the lesion edges into small amounts of •OH, thereby inducing the polarization of M1‐type macrophages (Figure 7Ab). This polarization of macrophages enhances the phagocytosis of fragmented biofilms and planktonic bacteria while stimulating the secretion of pro‐inflammatory cytokines (Figure 7Ac,d). Consequently, this strategy facilitates sterilization and prevents the occurrence of acute infections. Remarkably, this antimicrobial approach was independent of antibiotics, had minimal side effects, and offered precise biofilm elimination at the infection site, making it a promising strategy for combined CDT and immunotherapy. It is important to note that the limited levels of endogenous H_2_O_2_ at the infection site pose a significant challenge to the sterilization efficacy of CDT. The incorporation of Gox within the chemodynamic agents offers the advantage of in situ H_2_O_2_ generation during infection. When Gox and a chemodynamic agent is coloaded in a therapeutic system, it acts upon the glucose present in the BME, generating H_2_O_2_ and gluconic acid. Consequently, the combination of Gox and Fenton‐like catalytic reactions significantly enhances the sterilization effect of CDT. The replenished H_2_O_2_ provides a sustained source for CDT reactions, while the lowered pH creates a more favourable environment for the generation of ROS. This synergistic effect contributes to the effectiveness of CDT in eradicating bacterial infections.^[^ ^44^, ^67^ ^]^ Su et al. developed a nanobionics catalyst with a bilayer structure, known as MACG, for combating peri‐implant biofilm infections (Figure 7B).^[^ ^77^ ^]^ The preparation involved loading an activatable photothermal agent (ABTS) into the MOF of MIL‐100 and anchoring Cu^2+^ in the pore channels of MIL‐100. The surface of MIL‐100 was then successfully sealed, and its internal pore channels were closed through the growth of CuBTC (BTC, benzene‐1,3,5,‐tricarboxylic acid), and then Gox was loaded onto the CuBTC surface. In the acidic BME, CuBTC degrades, leading to the release of Cu^2+^ ions and Gox. Cu^2+^ ions, in turn, participate in the Fenton reaction with H_2_O_2_, generating highly toxic •OH and effectively killing the biofilm. Furthermore, Cu^2+^ ions contribute to the reduction of GSH content in the BME, thereby limiting the consumption of •OH. Meanwhile, Gox facilitates the decomposition of glucose in the BME, producing gluconic acid and H_2_O_2_. The presence of gluconic acid lowered the pH of the biofilm microenvironment, promoting the degradation of CuBTC. Additionally, the produced H_2_O_2_ serves as a raw material for the Fenton reaction. Importantly, the endogenous H_2_O_2_ present in the biofilm microenvironment oxidized ABTS to ox‐ABTS, resulting in enhanced near‐infrared light absorption efficiency and photothermal conversion efficiency. MACG not only demonstrates its efficacy in combating biofilm infections but also exhibits immunomodulatory properties by stimulating macrophages to transition to the M1 phenotype, which plays a role in re‐establishing a pro‐inflammatory immune microenvironment at the site of bacterial infection. By combining CDT with immunotherapy, MACG effectively triggers itself photothermal activity and generates highly toxic •OH at the infection site, thus reversing the immunosuppressive microenvironment associated with biofilm infections. Importantly, the experimental results demonstrated the favourable biosafety of MACG, as evidenced by the stable body weight of mice injected with MACG and the absence of observable pathological changes in the mice's organs. The combination of CDT and immunotherapy presents synergistic effects in the treatment of bacterial infections. The addition of immunotherapy enhances the bactericidal activity through the collaborative action of M1‐type macrophages and highly toxic •OH generated during the CDT process. In addition, the •OH is capable of disrupting the biofilm structure and overcoming the immune barrier posed by bacteria, facilitating direct contact between immune cells or their secreted products and the bacteria, thereby enhancing the effectiveness of immunotherapy.

### 4.7 CDT‐ferroptosis

**FIGURE 8:**
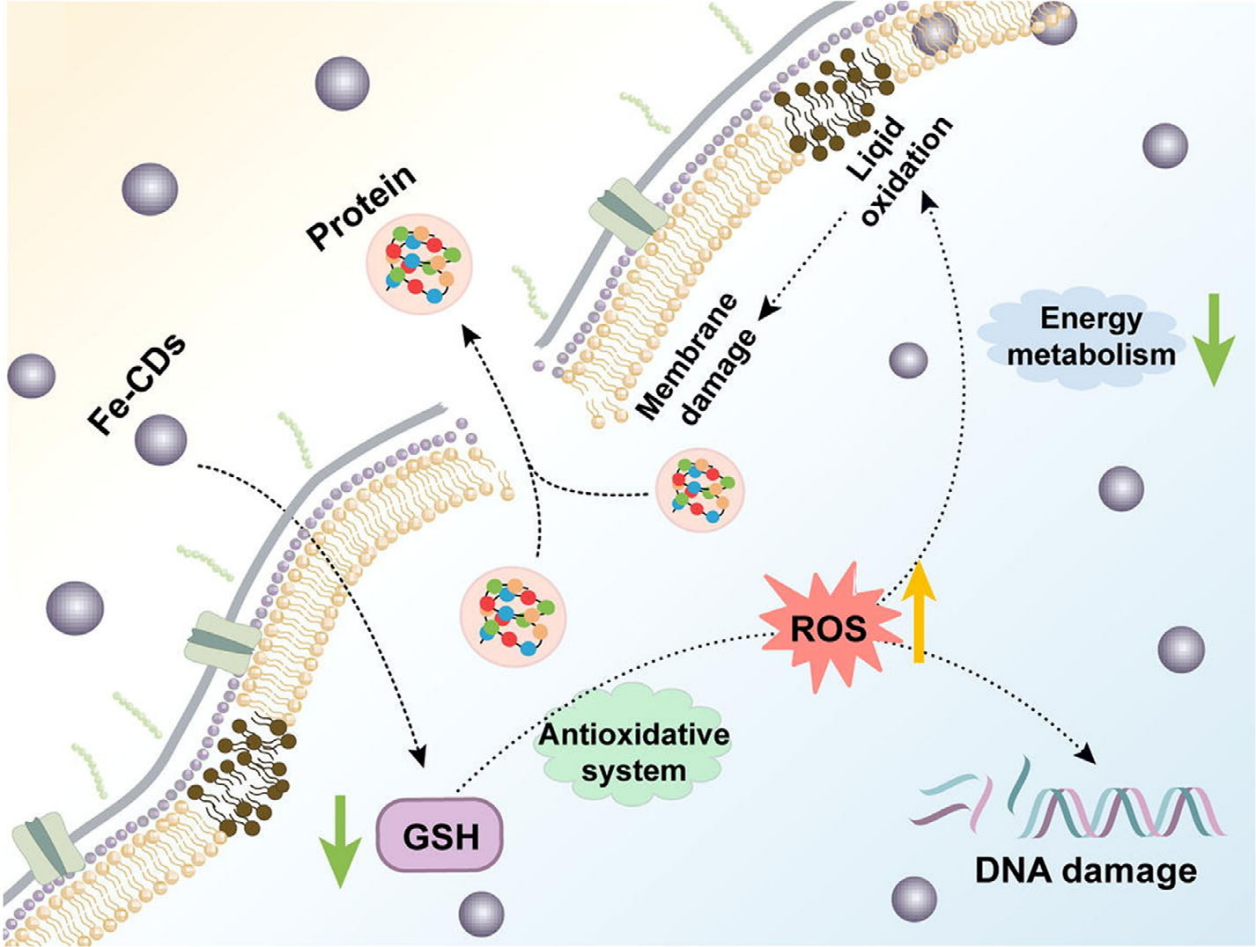
Fe‐CDs for combined CDT/Ferroptosis antimicrobials. Reproduced with permission.^[^ ^80^ ^]^ Copyright 2023, Elsevier Ltd.

Ferroptosis is a different type of programmed cell death. It consumes large amounts of GSH through the accumulation of iron in the cells, which in turn inactivates the GSH peroxidase (GPX).^[^ ^78^ ^]^ The ROS produced by the Fenton reaction can induce the peroxidation of unsaturated fatty acids, further driving cells to ferroptotic death.^[^ ^79^ ^]^ Due to the accumulation of iron ions and GSH depletion, an enhanced therapeutic effect will be obtained. Huang et al. used urea, FeCl_3_, citric acid, water, and a microwave oven to synthesize iron‐doped CDs (Fe‐CDs) for antibacterial application (Figure 8).^[^ ^80^ ^]^ When the bacteria were treated with Fe‐CDs, the permeability of the bacterial membrane increased, and then the bacterial membrane was damaged, resulting in the leakage of intracellular materials and bacterial death. In addition, after Fe‐CDs treatment, the ROS content within the bacteria is significantly elevated, and the excess of ROS leads to the disruption of the balance of the antioxidant system within the bacteria. At the same time, the content of iron ions within the bacteria is significantly elevated, and the accumulated iron ions react with the bacterial metabolite H_2_O_2_ via the Fenton reaction. The ROS produced by the Fenton reaction and the oxidative stress within bacteria induced by Fe‐CDs, which in turn enhance the antibacterial capacity of CDT. Moreover, the lipid peroxides formed by the oxidation of ROS affect the stability as well as the fluidity of the membrane, and membrane instability eventually develops into pores that lead to the outflow of cytoplasmic contents and bacterial death. The experimental results showed that genetic material was destroyed by Fe‐CDs, which in turn affects the replication and translation of bacterial nucleic acids, and other processes. Thus, Fe‐CDs with CDT and Ferroptosis combined therapy becomes a promising treatment for bacterial infections.

During bacterial ferroptosis, the enrichment of iron ions can consume a large amount of GSH while providing powerful conditions for the occurrence of the Fenton reaction. The combination of CDT and ferroptosis can generate a large amount of ROS to kill bacteria, which provides a new route for the treatment of bacterial infections.

## 5 CONCLUSION

With the increasing prevalence of bacterial resistance due to the extensive use of antibiotics, there is a critical need for the development of more efficient and convenient methods for bacterial treatment.^[^ ^81^ ^]^ In this regard, CDT has emerged as a promising approach and has garnered significant attention among researchers.^[^ ^82^ ^]^ This work provides a comprehensive review of strategies aimed at enhancing the therapeutic effectiveness of CDT, including increasing the H_2_O_2_ content at the infection site, inhibiting GSH overexpression, and reducing the pH level of the infected tissue. Additionally, various combined therapies involving CDT, such as CDT‐PTT, CDT‐PDT, CDT‐SDT, CDT‐starvation therapy, CDT‐antimicrobial peptide, and CDT‐immunotherapy, have been introduced.^[^ ^20c^ ^]^ These approaches have demonstrated substantial improvements in antimicrobial efficacy while minimizing toxic side effects.

## 6 OUTLOOK

Despite the rapid development of CDT in recent years, several crucial issues still require to be investigated.^[^ ^83^ ^]^

First, as the eventual clinical application of chemodynamic nanoagents is anticipated, the biosafety of these nanomaterials must be thoroughly evaluated.^[^ ^84^ ^]^ Although current studies indicate minimal biotoxicity of prepared chemodynamic nanoagents, future research should focus on reducing potential toxic effects while enhancing therapeutic efficacy.^[^ ^43^, ^85^ ^]^ Second, a deeper understanding of the operation mechanism of CDT is needed.^[^ ^86^ ^]^ While Fenton‐like reactions for generating •OH have been extensively studied, the microenvironment of bacterial infections poses challenges to •OH production due to factors such as pH, GSH content, and H_2_O_2_ levels. Therefore, continuous efforts should be devoted to improving the rate of Fenton‐like reactions to ensure the abundant production of •OH at the bacterial infection site for effective bacterial eradication.^[^ ^87^ ^]^ In addition, uncovering the ROS generation mechanism of CDT is crucial for achieving efficient bacterial elimination in infected tissues.^[^ ^88^ ^]^

Third, enhancing the targeting capabilities of chemodynamic nanoagents is essential for precise antimicrobial treatment.^[^ ^89^ ^]^ By combining CDT with new technologies and new nanomaterials, the targeting of chemodynamic agents can be improved, enabling selective bacterial eradication at the infection site while minimizing damage to healthy tissue. Furthermore, a real‐time assessment of treatment effectiveness should be conducted to verify and validate its efficacy.^[^ ^73^ ^]^

Lastly, the development of chemodynamic nanoagents with multimodal therapeutic properties is gaining significant interest.^[^ ^90^ ^]^ It is evident from current research that CDT in combination with other treatments can significantly improve efficacy and thus provide effective killing of bacteria. However, combined therapeutic strategies are still in their infancy and face many problems that need to be solved, including the integration of diagnosis and treatment, ways to improve the effectiveness of treatment, and reduction of toxic side effects.^[^ ^91^ ^]^ And the advantages and disadvantages of different combined therapeutic strategies were summarized briefly in Table 2.

**TABLE 2:** The advantages and disadvantages of combination therapy.

Combination therapy	Advantages	Disadvantages
CDT‐PTT	Hyperthermia‐enhanced Fenton reaction rate, high spatiotemporal selectivity	Limited tissue penetration, inducing thermal damage to surrounding tissues
CDT‐SDT	Improving ROS content, unlimited tissue penetration, high spatiotemporal selectivity, unlimited tissue penetration	Oxygen requirement
CDT‐PDT	Improving ROS content, relieving biofilm hypoxia, high spatiotemporal selectivity,	Limited tissue penetration, inducing oxidative damage to surrounding tissues
CDT‐immunotherapy	Reducing infection recurrence by enhancing immune reaction	Inducing damage to immune system
CDT‐starvation therapy	decreasing the pH value in the infected tissue, providing H_2_O_2_ for CDT,	The instability of natural enzyme (e.g., GOx)
CDT‐antibacterial peptide	The ability to target bacteria	The instability of natural of natural antibacterial peptide AMPs
CDT‐ferroptosis	Enhancing oxidative damage by interfering cellular antioxidant performance	Time‐consuming

Continuous in‐depth research on novel chemodynamic nanoagents offers promising prospects for antibacterial applications. While current studies encounter various challenges, advancements in science and technology will likely contribute to the future application of emerging CDT nanoagents in clinical practice.

## CONFLICT OF INTEREST STATEMENT

The authors declare no conflicts of interest.
